# Secondary Outcomes of Implemented Depression Prevention in Adolescents: A Randomized Controlled Trial

**DOI:** 10.3389/fpsyt.2021.643632

**Published:** 2021-02-23

**Authors:** Karlijn W. J. de Jonge-Heesen, Sanne P. A. Rasing, Ad A. Vermulst, Ron H. J. Scholte, Kim M. van Ettekoven, Rutger C. M. E. Engels, Daan H. M. Creemers

**Affiliations:** ^1^GGZ Oost Brabant, Boekel, Netherlands; ^2^Erasmus School of Social and Behavioural Sciences, Erasmus University Rotterdam, Rotterdam, Netherlands; ^3^Behavioural Science Institute, Radboud University Nijmegen, Nijmegen, Netherlands; ^4^Praktikon, Nijmegen, Netherlands

**Keywords:** cognitive behavior therapy, depressive symptoms, perfectionism, somatic symptoms, anxiety, suicidality, prevention, adolescence

## Abstract

Our most recent RCT provides evidence that indicated depression prevention is effective in reducing depressive symptoms in adolescents when implemented in the school community. In the present study we further test the potential effects of this prevention approach on symptoms related to depression: anxiety, suicidality, somatic symptoms, and perfectionism. We conducted exploratory analyses in 130 adolescents with elevated depressive symptoms aged between 12 and 16 years old (*M* = 13.59; *SD* = 0.68; 63.8% girls) who were randomly assigned to the experimental (OVK 2.0) or active control condition (psycho-education). Self-reported anxiety, suicidality, somatic symptoms, and perfectionism were assessed at pretest, post intervention, as well as 6- and 12-months follow-up. Latent growth curve analyses revealed that there was a significant decrease in anxiety in both conditions and that this decrease was significantly larger in the intervention condition than in the control condition. Somatic symptoms and socially prescribed perfectionism decreased significantly in the intervention condition and suicidality decreased significantly in the control condition. Yet there was no difference in decrease in suicidality, somatic symptoms, and perfectionism between the two conditions. This study suggest that screening on depressive symptoms and providing a CBT depression prevention program for adolescents with elevated depressive symptoms, can decrease comorbid symptoms of anxiety and therefore ensure better outcomes. We discuss the clinical implications as well suggestions for future research.

**Clinical Trial Registration:** The study is registered in the Dutch Trial Register for RCTs (NTR5725). Date registered: 11th of March 2016.

## Introduction

The number of adolescents experiencing depression is substantial, with ~15.5% of adolescents experiencing depression between the ages of 11 and 19 (1). Moreover, these rates have increased in recent years, with a growing number of adolescents with untreated depression (2). The consequences of depression are tremendous, especially in adolescence. Important developmental processes take place in this phase of life, for instance the development of positive relationships and the maturation of skills that are important for life and work (3). It is therefore not surprising that the experience of depression in this developmental period is associated with several poor outcomes such as failure to complete secondary school, unemployment, and substance misuse (4, 5). Considering the negative outcomes, the prevention of depression should be a priority.

Several meta-analyses have shown that prevention programs could be effective in the prevention of depression, with the largest effect sizes for programs designed for adolescents who already have elevated depressive symptoms (6–10). Yet the implementation of these programs seems to suffer from practical barriers such as lack of communication between researchers and practitioners, poor financing, and interventions that are too complex, costly, or narrowly focused (11, 12). Until recently, it has been unclear whether the prevention effects that were found would remain when preventive interventions are implemented on a large scale.

Our most recent randomized controlled trial (RCT) about an integrated depression prevention approach (STORM: Strong Teens and Resilient Minds) examined the effectiveness of indicated prevention in reducing depressive symptoms in adolescents. This approach has a strong focus on collaboration between schools and (mental) health care partners and includes: (1) early screening for depressive symptoms and suicidal ideation, followed by clinical referral for students with acute suicidality; and (2) an indicated depression prevention program for adolescents with elevated depressive symptoms. The integration of STORM in the school community made it possible to examine the effectiveness of depression prevention under real life circumstances. In the RCT, the Cognitive Behavioral Therapy (CBT) based program entitled “Op Volle Kracht” 2.0 (OVK 2.0) was compared with psycho-education. The findings showed that OVK 2.0 was significantly more effective in reducing depressive symptoms than psycho-education 1 year after the prevention program, although it should be noted that depressive symptoms decreased in both conditions (13, 14).

These important findings are the basis from which to further unravel the potential effects of this program on other internalizing problems. It is possible that prevention strategies aimed at depression also affect other internalizing symptoms, suggesting that more adolescents with mental health needs might benefit from this prevention approach. Accordingly, the purpose of this study is to conduct exploratory analyses of the effect of indicated depression prevention on symptoms related to depression, which are: anxiety, suicidality, perfectionism, and somatic symptoms.

Anxiety, suicidality, somatic symptoms, and perfectionism are all strongly related to depressive symptoms and co-occur in a high degree (15–19). Moreover, they seem to share the same biomarkers, underlying mechanisms, and risk factors as depression, and might therefore respond similarly to a specific prevention approach (20). Despite the high comorbidity, in clinical practice it is not uncommon that these concepts cover up symptoms of depression. For example, headache and abdominal pain, which are the most frequent complaints in adolescents, are often triggered by stress and, when not acknowledged, could ultimately lead to symptoms of internalizing problems (21, 22). Also, adolescents high in perfectionism are often internally motivated to conceal internalizing symptoms, in fear of falling short of standards (23). This impedes the detection of underlying depressive symptoms, which is detrimental for several reasons, one of which is that untreated adolescent depression is related to a recurrence of symptoms in adulthood (24).

Although anxiety, suicidality, somatic symptoms, and perfectionism are related to depressive symptoms, it is unknown whether a prevention program aimed at depressive symptoms affects other symptoms too. Due to the high comorbidity and shared etiology, it could be expected that a decrease in depressive symptoms is associated with lower levels of other adverse outcomes. The outcomes of this study would add valuable information for further implementation as it is more efficient to implement interventions that also target coexisting problems. Although these analyses are largely exploratory, we hypothesized that prevention would lead to a reduction in symptoms. Specifically, we expect that adolescents who received OVK 2.0 would show larger reductions in anxiety, suicidality, somatic symptoms, and perfectionism than adolescents who received psycho-education.

## Methods and Materials

### Participants

As is described elsewhere (14), in this study a total of 5,222 adolescents in the second year of secondary schools were screened for depressive symptoms. Of the 5,222 adolescents, 469 had elevated depressive symptoms and these adolescents were approached for further study. Besides elevated depressive symptoms according to the screening [score ≥ 14; CDI-2; (25, 26)], inclusion criteria were: sufficient knowledge of the Dutch language, and age between 11 and 15 years old. Exclusion criteria were: presence of high suicidality, already undergoing CBT for mood problems, and absence of parental permission. Ultimately, 130 adolescents aged between 11 and 15 years old participated (*M* = 13.59; *SD* = 0.68; 63.8% girls). School levels varied between vocational training (45.4%) and pre-university training (19.2%). The majority of the participants were of Dutch origin (85.4%). After obtaining informed consent from adolescents and parents, participants were randomly allocated to OVK 2.0 (*n* = 66; the intervention condition) or psycho-education (*n* = 64; the control condition). Randomization was stratified on school level and was performed by an independent researcher. Participants completed online surveys at baseline (T1), after the intervention (T2), at 6-month follow-up (T3), and at 12-month follow-up (T4). After completion of each survey, participants received a gift voucher. More information about the participant flow is provided in Supplementary File 1, presenting a flow diagram of the study.

### Interventions

#### OVK 2.0

OVK has its origin in the Penn Resiliency Program [PRP; (27)], which was developed in the United States and proved to be effective as universal prevention within a school setting (28). In the Netherlands, OVK was investigated on several prevention levels, and it was concluding that the program was not effective in the prevention of depressive symptoms on a universal and selective level (29, 30). In a shortened protocol (8 lessons instead of 16), OVK was proved to be effective in adolescent girls with elevated depressive symptoms (31). Consequently OVK 2.0 is a modified version of the original OVK program based on the program that was used in the study of Wijnhoven et al. (31). The goal of OVK 2.0 is to teach adolescents how to recognize their thoughts and emotions, and how these are related with each other and with their behavior. The training was given in eight 1-h lessons in groups of three to eight adolescents, and the techniques in the training were based on CBT. Trainers had to fill in a checklist of exercises after each lesson to measure the treatment fidelity. Adherence to the protocol ranged from 74.6 to 94.7%. The study protocol and article presenting the main effects present more details about the content of the program and the background of the trainers (13, 14).

#### Psycho-Education

Psycho-education consisted of a brochure with information about depressive symptoms and two e-mails with advice and tips on how to decrease depressive symptoms. For example, adolescents were encouraged to continue doing activities that used to give them a positive feeling.

### Measures

*Anxiety* was measured with the State-Trait Anxiety Inventory [STAI; (32)]. We used the 20 items measuring state anxiety. Participants had to rate on a 4-point scale that ranged from 0 (almost never) to 3 (almost always) how they feel at the moment (e.g., “I feel nervous”). Cronbach's alpha ranged from 0.91 to 0.93 over the various assessment points.

*Suicidality* was measured with the VOZZ-Screen (33). This 10-item questionnaire assesses thoughts and actions about suicide, suicidal ideations, self-harm, and life. Items about life (e.g., “I feel worthless”) are rated on a 5-point scale ranging from 1 (I totally agree) to 5 (I totally disagree). Items about self-harm and suicide (e.g., “I attempted suicide”) are rated on a 5-point scale from 1 (never) to 5 (very often). Items about suicidal ideation in the past week (e.g., “I thought that suicide would be a solution for my problems”) are rated on a 5-point scale from 1 (never) to 5 (every day). Cronbach's alpha ranged between 0.79 and 0.81 over the various assessment points.

A sum score of 23 or above is an indication of a serious suicide risk. Adolescents who appeared to be at high risk for suicidality by a score of 23 or above or by filling in the item about suicide in the CDI-2 with “I want to end my life,” were seen by a professional of the public health service within the school. Subsequently, parents were informed, and eventual information about referrals were provided.

*Somatic symptoms* were measured with the Dutch version of the Children's Somatization Inventory [CSI; (34, 35)], consisting of 35 items on which participants had to rate on a 5-point scale from 0 (no suffering) to 4 (much suffering) to what extent they have been bothered by somatic symptoms in the past 2 weeks (e.g., “abdominal pain”). Cronbach's alpha was 0.92 at all timepoints.

*Perfectionism* was measured with the Dutch version of the Frost Multidimensional Perfectionism Scale [F-MPS; (36, 37)]. This questionnaire contains 35 items and six subscales of perfectionism: concern over mistakes, doubts, personal standards, organization, parental expectations, and parental criticism. Participants have to rate to what extent each statement fits them on a scale ranging from 1 (strongly disagree) to 5 (strongly agree). For the purpose of the present study, we only used the subscales concern over mistakes (e.g., “I hate being less than the best at things”), doubt about actions (e.g., “I usually have doubts about the simple everyday things I do”), and personal standards (e.g., “I set higher goals than most people”).

In line with the literature on perfectionism (19), we distinguished two factors in perfectionism: personal standards perfectionism (PS; sum score of personal standards, 7 items) and concerns about mistakes and doubts perfectionism (CMD; sum scores of concerns about mistakes and doubt about actions, 13 items). PS represents self-orienting perfectionism (setting unreasonably high standards and goals) and CMD represents socially prescribed perfectionism [doubts and excessive concern for mistakes; (36, 38)]. Cronbach's alpha ranged between 0.86 and 0.88 for PS and between 0.91 and 0.94 for CMD over the various assessment points.

### Strategy of Analyses

Data were analyzed with the statistical package Mplus version 7.2 (39). First, we used descriptive statistics and z-tests to analyze differences in the measured concepts at all timepoints. Next, we used Latent Growth Curve Models (LGCM) to test the longitudinal effectiveness of OVK 2.0 on secondary outcomes, according to the intent-to-treat principle. The Full Information Maximum Likelihood estimator [FIML; (40, 41)] was used to handle missing data under the condition that missings are at random. Little's MCAR test showed that completely missing at random was supported (χ^2^_[362]_ = 394.81, *p* = 0.113). Five participants were excluded from the analyses because of missing data at all four timepoints, two from the intervention condition and three from the control condition.

The procedure COMPLEX with the robust maximum likelihood estimator (MLR) was used to control for non-independence of the data because of nesting participants within the 13 schools. We used the following fit indices: Chi-square (*df*), the Root Mean Square of Approximation [RMSEA; values < 0.08 means acceptable fit; (42)], and the Comparative Fit Index [CFI; values > 0.90 means acceptable fit; (43)].

In the study for main effects of the RCT (14), a linear growth model for depressive symptoms was accepted above a quadratic one, because a quadratic model was overfitting the data (44). This was also the case for the secondary outcomes, and a linear growth model for each of the secondary outcomes was accepted as most adequate. Parameters were intercept (*i*; initial estimated level) and slope (*s*; estimated degree of change over time) as latent growth parameters, and time was coded in months (0, 3, 6, and 12 months). For anxiety, the linear model showed a fit of χ^2^_(12)_ = 33.35, *p* = 0.001, RMSEA = 0.169, CFI = 0.904. For suicidality, the fit of the model was χ^2^_(12)_ = 12.11, *p* = 0.437, RMSEA = 0.012, CFI = 0.999. For somatic symptoms, the fit of the model was χ^2^_(12)_ = 31.21, *p* = 0.002, RMSEA = 0.161, CFI = 0.888. The model fit of PS perfectionism was χ^2^_(12)_ = 34.75, *p* = 0.001, RMSEA = 0.175, CFI = 0.866. Finally, the model fit of CMD perfectionism was χ^2^_(12)_ = 11.28, *p* = 0.505, RMSEA = 0.000, CFI = 1.000. The fit of three models was acceptable for the CFI with values > 0.90, but two models had a CFI-value somewhat below 0.90. Additionally, the fit for three models was less acceptable for the RMSEA (the models of anxiety, somatic symptoms, and PS perfectionism). However, for small samples cutoff values of 0.10 for RMSEA are too restrictive (45), and acceptable models might be over-rejected (46). Moreover, poor global fit indices (CFI and RMSEA) can be misleading: they may still be consistent with a good approximation of individual growth curves (47). Therefore, these models were accepted.

Next, we used the χ^2^ difference test to test differences in intercept between the intervention and control condition, by comparing the χ^2^ value of the unconstrained model with the χ^2^ value of the growth model where both intercepts were constrained to be equal. A significant difference in intercept was indicated when the χ^2^ value significantly differed between the conditions. For testing differences in slope, the testing procedure was repeated by comparing the equal intercept constrained model with the equal intercept and equal slope model.

## Results

As emerged from the screening, 469 adolescents reported elevated depressive symptoms. Of these adolescents, 130 participated in our study. The percentage of adolescents completing the surveys at baseline (T1), post-intervention (T2), 6-month (T3) follow-up, and 12-month (T4) follow-up were 88.5, 71.5, 80.0, and 80.0%. The descriptive statistics and test results of the comparison between intervention and control condition for all secondary outcomes are presented in Table 1. No significant differences between the intervention and control condition in suicidality, somatic symptoms, and CMD were found. Anxiety differed with marginal significance between the conditions at T4, with higher means in the control condition. In addition, PS differed significantly between the conditions at T2 and at T4, with higher means in the control condition. Correlations between the outcome variables and depressive symptoms are presented in Supplementary File 2.

**Table 1:** Means, standard deviations, and z-values for differences on anxiety, suicidality, somatic symptoms, and perfectionism (PS and CMD) between the intervention and control conditions.

	**Intervention condition (*****N*** **=** **64)**		**Control condition (*****N*** **=** **61)**			
	***M***	***SD***	***M***	***SD***	***z*-value**	***P***
Anxiety T1	42.73	10.21	42.51	11.18	0.13	0.896
Anxiety T2	38.72	11.15	39.38	11.35	−0.27	0.786
Anxiety T3	38.48	11.96	40.29	11.79	−1.17	0.241
Anxiety T4	34.65	11.07	38.50	10.58	−1.92	0.055
Suicidality T1	17.92	4.77	19.40	6.42	−1.23	0.219
Suicidality T2	17.76	6.22	19.24	6.50	−1.62	0.105
Suicidality T3	16.83	5.56	18.61	6.52	−1.70	0.089
Suicidality T4	16.70	5.76	18.06	5.90	−1.75	0.080
Somatic symptoms T1	20.01	13.76	22.24	18.63	−0.60	0.547
Somatic symptoms T2	19.22	16.49	18.17	17.03	0.44	0.660
Somatic symptoms T3	16.46	14.15	19.82	16.85	−1.21	0.226
Somatic symptoms T4	16.87	15.78	18.51	16.95	−0.37	0.715
PS perfectionism T1	15.19	7.15	15.24	6.26	−0.08	0.935
PS perfectionism T2	13.18	5.41	15.30	6.90	2.71	0.007
PS perfectionism T3	14.33	6.08	15.53	7.33	−1.58	0.114
PS perfectionism T4	13.80	5.61	15.34	6.94	−2.38	0.017
CMD perfectionism T1	26.97	10.91	27.88	10.92	−0.36	0.715
CMD perfectionism T2	24.14	10.46	25.38	11.48	−1.17	0.243
CMD perfectionism T3	25.15	11.36	26.56	12.93	−0.84	0.400
CMD perfectionism T4	22.93	11.07	25.53	10.61	−1.65	0.099

## Latent Growth Curve Modeling

First, we examined the linear growth models of anxiety, suicidality, somatic symptoms, PS perfectionism, and CMD perfectionism for the intervention and control conditions. The results of these analyses are presented in Table 2. Besides the intercepts and slopes, the fit measures of the baseline models are also described in this table. The results show that slopes are significant for anxiety, showing that anxiety decreased over time in both conditions. The significant negative slopes for somatic symptoms and CMD perfectionism in the intervention condition indicate a decrease over time as well. Furthermore, suicidality decreased significantly in the control condition and showed a decreasing trend in the intervention condition.

**Table 2:** Results of latent growth curve analyses for the five outcome variables.

	**Intervention group**				**Control group**				**Fit measures of baseline model**				**Test between groups**			
													**For intercepts**		**For slopes**	
	**i**	***p***	**s**	***p***	**i**	***p***	**s**	***p***	**χ^**2**^(12)**	***p***	**CFI**	**RMSEA**	***Δχ*^**2**^(1)**	***p***	***Δχ*^**2**^ (1)**	***p***
Anxiety	41.91	0.000	−0.62	0.000	41.40	0.000	−0.24	0.001	33.35	0.001	0.904	0.169	0.08	0.777	5.76	0.016
Suicidality	17.84	0.000	−0.10	0.065	19.41	0.000	−0.11	0.001	12.11	0.437	0.999	0.012	2.42	0.120	0.26	0.610
Somatic symptoms	19.29	0.000	−0.21	0.036	20.62	0.000	−0.18	0.070	31.21	0.002	0.888	0.161	0.23	0.632	0.12	0.729
PS perfectionism	13.69	0.000	0.02	0.682	15.28	0.000	0.02	0.778	34.75	0.001	0.866	0.175	1.74	0.187	0.53	0.467
CMD perfectionism	26.15	0.000	−0.27	0.005	27.45	0.000	−0.16	0.150	11.28	0.505	1.000	0.000	0.44	0.507	1.10	0.294

**Figure 1:**
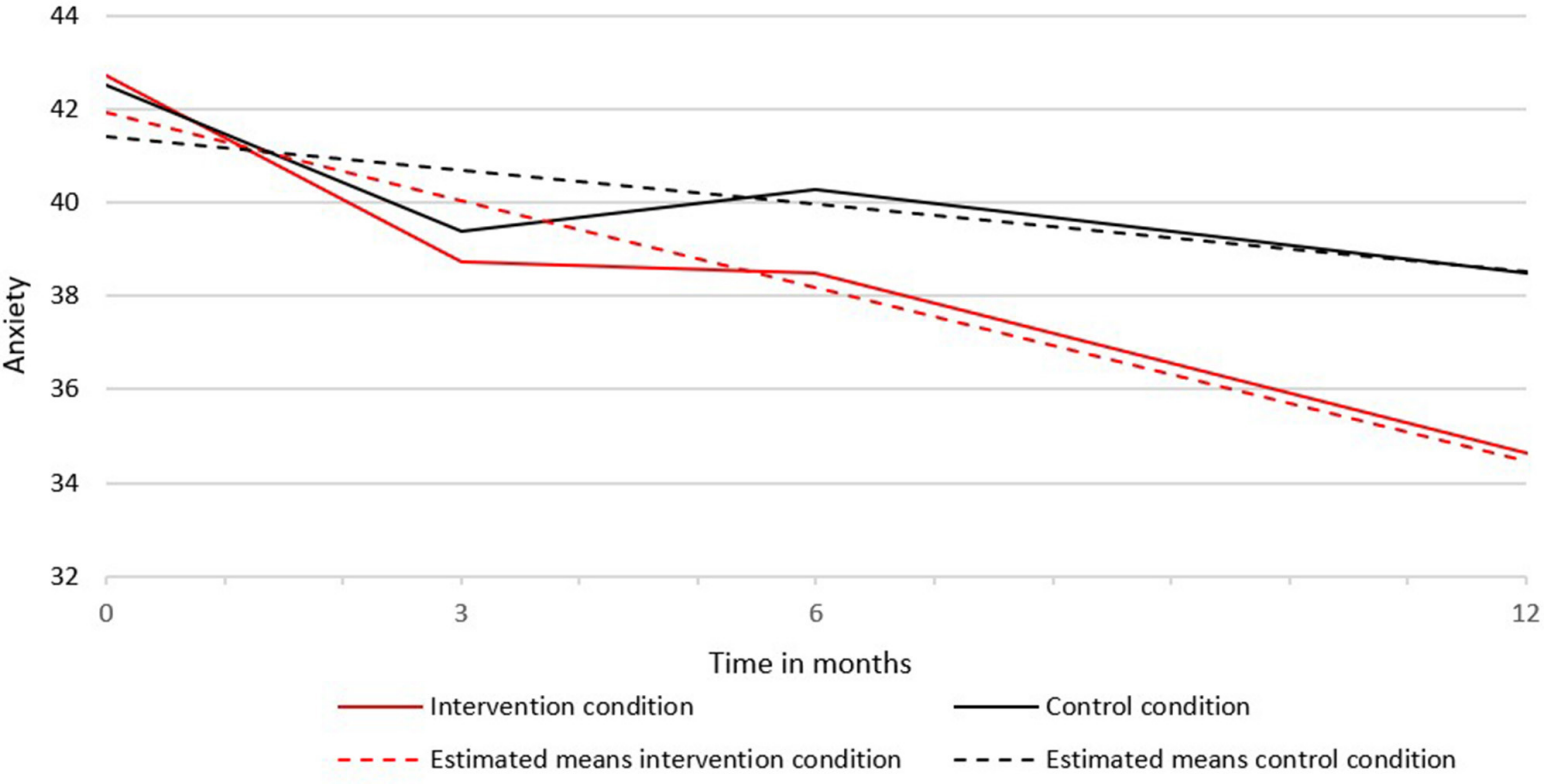
Mean scores of anxiety over time in the intervention and control condition.

Second, we tested whether intercept and slopes differed between the intervention and control condition (last four columns in Table 2). For anxiety only, the Chi-square difference tests between groups showed that the slopes in the intervention and control group were significantly different (see Table 2). The decrease in anxiety in the intervention condition (s = −0.62) was stronger than in the control condition (s = −0.24). Figure 1 shows the course of anxiety in the intervention and control condition.

## Discussion

This study examined the effectiveness of depression prevention on anxiety, suicidality, somatic symptoms, and perfectionism in an implemented depression prevention approach for adolescents with elevated depressive symptoms. The findings from the present study showed that anxiety decreased significantly in both conditions and that the decrease was significantly greater in the intervention condition than the control condition. Furthermore, somatic symptoms and concerns about mistakes and doubts perfectionism decreased significantly in the intervention condition, and suicidality decreased significantly in the control condition. However, the decreases in somatic symptoms, concerns about mistakes and doubts perfectionism, and suicidality did not significantly differ between the two conditions. In addition to the significant effect on depressive symptoms (14), these findings show that the integrated prevention approach in this study might have broader effects than targeting depressive symptoms.

The significant effect of the depression prevention program on anxiety is encouraging, considering the evidence that 10–50% of the adolescents have comorbid levels of depression and anxiety (48, 49), and that the presence of comorbid anxiety predicts a severity in depressive symptoms (50, 51). In addition, the presence of both depression and anxiety predicts worse outcomes (e.g., increased risk of recurrence or poor treatment response) than either of these alone (52–54). The present study suggests that screening for depressive symptoms and providing a CBT depression prevention program for adolescents with elevated depressive symptoms can decrease comorbid symptoms of anxiety, and therefore has the potential to ensure better outcomes.

This finding is in line with research showing that CBT is effective for a wide range of emotional problems, including symptoms of anxiety (55). Although CBT programs for anxiety and depression vary in the strategies that are included, they share the same focus, which is cognitive restructuring by teaching the interplay between thoughts, feelings, and behaviors. Moreover, the CBT techniques might focus on the fundamental cognitive distortions that underlie both anxiety and depression (56). For example, the fear of rejection or the belief that one is not capable enough can cause both depressive symptoms and symptoms of anxiety. This overlap in techniques and focus might account for the significant effect of depression prevention on anxiety (20, 57).

However, the effect of depression prevention on anxiety is in contrast with [Garber et al. (56)], who tested in a meta-analytic review the cross-over effects of anxiety programs on depressive symptoms, and of depression programs on symptoms of anxiety. They found crossover effects for both depression and anxiety in treatment programs but not in targeted prevention programs, concluding that treatments for anxiety and depression may have broader effects than just the target they aimed at, but that prevention programs do not. Yet the review was focused on effects directly after treatment, which might underestimate prevention effects, as in our RCT significant effects were found 1 year after the program. Also, the mean level of depressive symptoms in our sample was near the level of clinical symptoms (*M* = 15.76, clinical symptom level ≥ 14), which might indicate that our findings are more comparable with treatment effects.

Still, the fact that despite the high comorbidity with depressive symptoms and their shared etiology, CBT depression prevention was not significantly more effective in the reduction of suicidality, somatic symptoms, and perfectionism than psycho-education, is thought-provoking. One explanation might be found in the content of the prevention program, which might not be sufficient in targeting these symptoms. Considering the content and therapeutic elements in interventions that target suicidality, somatic symptoms, and perfectionism, there are specific techniques that were not included in our prevention approach. For example, studies on adults support the use of CBT in the treatment of somatic symptoms, with 6–16 sessions of CBT leading to a reduction in symptoms (58). Yet these treatments include, besides the traditional CBT techniques, techniques that are more body oriented, such as relaxation techniques, mindfulness, guided imagery, and techniques that deal with specific somatic symptoms (59). Mindfulness is also suggested by researchers as an effective technique for treating perfectionism, in particular by learning to disengage from repetitive negative thinking (60). Furthermore, programs aimed at the reduction of suicidality contain interventions that differ from traditional CBT programs, such as techniques to increase help-seeking behavior, social support, and safety behavior (61). So, although CBT might have some benefits for these symptoms, they might require alternative or at least additional techniques.

According to this interpretation, the fact that not all comorbid problems respond to the same prevention strategy has some important implications for future research as well as for clinical practice. Since our main findings show that there is a substantial group of adolescents who did not respond to the CBT prevention in terms of a decrease in depressive symptoms (61.7%), we need to examine how prevention effects can be maximized. It is possible that there is a group of adolescents who did not respond to CBT prevention because of comorbid symptoms that impede the prevention effect. Arguing that the presence of certain symptoms, for instance perfectionism, calls for another intervention might also suggest that CBT is less effective in reducing depressive symptoms when there is comorbid perfectionism. Although future research should disentangle this further, more knowledge about the group of non-responders might lead to a more personalized prevention approach.

## Strengths and Limitations

The most important strengths of this study are the longitudinal design, the use of an active control group, and the implementation of preventive interventions in school communities. These strengths made it possible to examine the effectiveness of OVK 2.0 under real life conditions and to make substantial conclusions about the effectiveness. Also, the results are generalizable as the sample include both boys and girls from different school levels. Still, this study has some limitations. Although the sample was large enough to examine the effect on the outcome variables, it was insufficient to examine the effect on outcomes variables when controlling for depressive symptoms or as moderators in the effect on depressive symptoms. Such analyses would provide more information about the underlying mechanism of prevention and the additional effect of prevention on related symptoms when accounting for depressive symptoms. In addition, only 27% of the adolescents who emerged from the screening were willing to participate in the study, and therefore, selection bias must be considered [see also (14)]. Other limitations are the reliance on self-reports only, which might have caused socially desirable behavior, the lack of measurement of the fidelity of psycho-eduction, and the possible performance and assessment biases as allocation was not concealed. Finally, randomization was carried out on school level, which limited the random allocation of adolescents.

## Conclusions

The findings of the present study show that integrated depression prevention seems to be effective in reducing symptoms of anxiety in adolescents with elevated depressive symptoms. Although these symptoms frequently co-occur with depressive symptoms and share the same risk factors, we argue that additional techniques are necessary to target these problems. Regarding suicidality, we recommend future prevention studies to continue monitoring the effect of prevention programs on symptoms of suicidality (with appropriate risk management). Although just a small number of adolescents with suicidal ideation proceed to make an actual suicide attempt, the consequences for the environment are tremendous and we are obliged to do everything we can to decrease the number of suicides at this young age.

In conclusion, given the high prevalence rates of depression in adolescents and the poor outcomes when there is comorbid anxiety, these findings are hopeful. Therefore, this study provides further support for the implementation of an implemented prevention approach in which adolescents with elevated risk for depression are identified and offered an evidence-based prevention program to reduce the risk of developing depression or other negative outcomes.

## Data Availability Statement

The data for the current study is not publicly available due to the containing information that could compromise research participant privacy, but they are available from the corresponding author upon reasonable request.

## Ethics Statement

The studies involving human participants were reviewed and approved by CMO Arnhem-Nijmegen. Written informed consent to participate in this study was provided by the participants and participants' legal guardian/next of kin.

## Author Contributions

KJ-H, KE, SR, DC, and RE conceptualized and contributed to the design of the current study. KJ-H and KE were responsible for the coordination of the data collection. KJ-H wrote all the sections in the manuscript. SR reviewed and revised all sections of the manuscript. AV assisted in the planning, execution of data analyses, and description of the results. DC, RS, and RE all helped to draft the manuscript by providing feedback. All authors have made substantive intellectual contributions to the paper, read and approved the final manuscript.

## Conflict of Interest

The authors declare that the research was conducted in the absence of any commercial or financial relationships that could be construed as a potential conflict of interest.
