# Supplementary material for: Secondary Outcomes of Implemented Depression Prevention in Adolescents: A Randomized Controlled Trial
Source: Front Psychiatry. 2021 Feb 23;12:643632. doi: 10.3389/fpsyt.2021.643632 (PMC7940696; doi:10.3389/fpsyt.2021.643632)
Supplement: Supplementary file 1 [file Table_1.DOCX]

**Additional file 1.** Correlations between Study Variables and Depressive Symptoms.

|  | Depressive symptoms T1 | Depressive symptoms T2 | Depressive symptoms T3 | Depressive symptoms T4 |
| --- | --- | --- | --- | --- |
| Anxiety T1 | 0.73^***^ | 0.40^***^ | 0.38^***^ | 0.39^***^ |
| Anxiety T2 | 0.49^***^ | 0.75^***^ | 0.59^***^ | 0.62^***^ |
| Anxiety T3 | 0.44^***^ | 0.53^***^ | 0.74^***^ | 0.59^***^ |
| Anxiety T4 | 0.39^***^ | 0.58^***^ | 0.64^***^ | 0.73^***^ |
| Suicidality T1 | 0.65^***^ | 0.47^***^ | 0.42^***^ | 0.37^***^ |
| Suicidality T2 | 0.46^***^ | 0.68^***^ | 0.57^***^ | 0.50^***^ |
| Suicidality T3 | 0.54^***^ | 0.68^***^ | 0.74^***^ | 0.61^***^ |
| Suicidality T4 | 0.48^***^ | 0.60^***^ | 0.65^***^ | 0.70^***^ |
| Somatic symptoms T1 | 0.50^***^ | 0.31^**^ | 0.23^*^ | 0.18 |
| Somatic symptoms T2 | 0.43^***^ | 0.63^***^ | 0.53^***^ | 0.45^***^ |
| Somatic symptoms T3 | 0.51^***^ | 0.61^***^ | 0.65^***^ | 0.54^***^ |
| Somatic symptoms T4 | 0.41^***^ | 0.49^***^ | 0.48^***^ | 0.45^***^ |
| PS perfectionism T1 | 0.25^**^ | 0.12 | 0.17 | 0.15 |
| PS perfectionism T2 | 0.23^*^ | 0.29^**^ | 0.28^*^ | 0.26^*^ |
| PS perfectionism T3 | 0.33^**^ | 0.33^**^ | 0.50^***^ | 0.36^***^ |
| PS perfectionism T4 | 0.24^*^ | 0.30^**^ | 0.36^***^ | 0.32^**^ |
| CMD perfectionism T1 | 0.53^***^ | 0.33^**^ | 0.31^**^ | 0.25^*^ |
| CMD perfectionism T2 | 0.33^**^ | 0.58^***^ | 0.48^***^ | 0.42^***^ |
| CMD perfectionism T3 | 0.36^***^ | 0.50^***^ | 0.68^***^ | 0.50^***^ |
| CMD perfectionism T4 | 0.31^**^ | 0.50^***^ | 0.57^***^ | 0.59^***^ |

^*^*p* < .05; ^**^*p* < .01; ^***^*p* < .001
