# Supplementary figures and images for: Secondary Outcomes of Implemented Depression Prevention in Adolescents: A Randomized Controlled Trial
Source: Front Psychiatry. 2021 Feb 23;12:643632. doi: 10.3389/fpsyt.2021.643632 (PMC7940696; doi:10.3389/fpsyt.2021.643632)

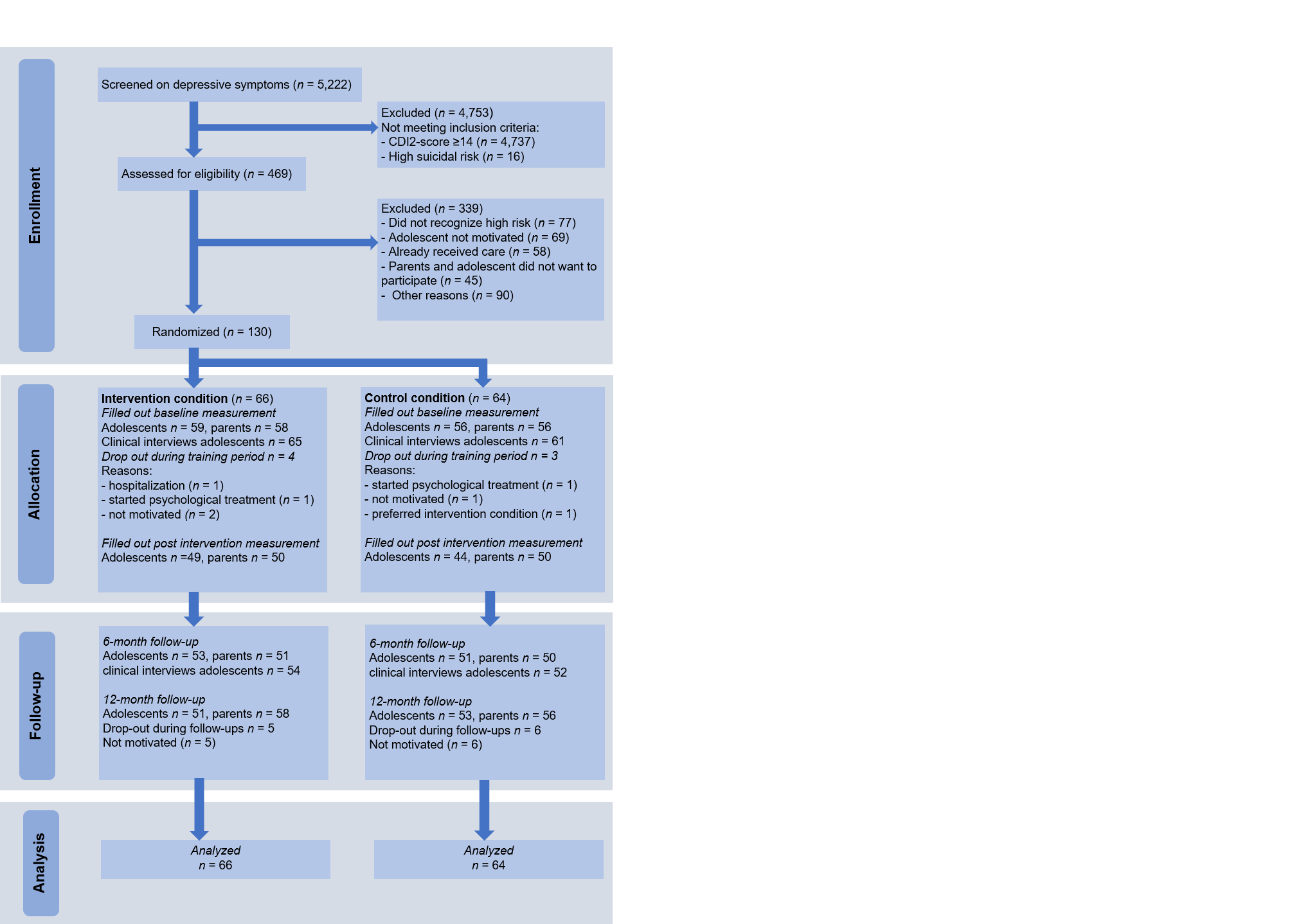

Supplement: Supplementary file 2 [file Image_1.PNG]
